# Deliberations on Unconscious thought Theory

**DOI:** 10.3389/fpsyg.2012.00350

**Published:** 2012-09-25

**Authors:** Jeffrey Chrabaszcz, Michael Dougherty

**Affiliations:** ^1^Department of Psychology, University of Maryland at College ParkCollege Park, MD, USA

Two central assumptions of unconscious thought theory (UTT) are the capacity assumption and the weighting assumption. The capacity assumption states that unconscious thought (UT) is unconstrained by cognitive capacity, thereby enabling people to make capacity-free choices when information can be aggregated unconsciously. The weighting assumption states that the unconscious is better able to weight various attributes by their relative importance. Ashby et al. (2011) present evidence challenging both of these assumptions. While the authors caution against overzealously applying UTT when making important decisions, they do not explicitly question the central claim of UTT: that an unconscious mode of thought exists and operates differently from conscious processing of information (Dijksterhuis and Nordgren, 2006). Nevertheless, Ashby et al.’s findings should motivate us to ask, first, is UTT a psychologically plausible theory of choice? And second, how can past work shown to support UTT, and the current work shown to contradict the model, be reconciled? We believe that the Ashby et al. paper begins to address both of these questions.

Is UTT a psychologically plausible model of choice? A good starting point to evaluating the plausibility of any theory is to establish its *a priori* probability (formally, a Bayesian prior). The hypothesis that one can make better choices without explicitly thinking about the options is indeed a counter-intuitive and exciting possibility. But, with counter-intuitive theories comes skepticism in the form of low prior probabilities (cf. Wagenmakers et al., 2011), and therefore the need for strong experimental evidence to change the skeptical Bayesian’s mind. Although Ashby et al. (2011) show some small advantages for UT for picking out the gamble with the highest expected value (EV) in some experiments, the effect is neither strong nor consistent across experiments. For example, in Experiment 3 participants in the UT condition did nominally worse than participants who were asked to deliberate without information, and significantly worse than those participants who deliberated with information. Moreover, across all three experiments, participants who viewed the relevant information during a delay period either performed equivalent to or better than the unconscious deciders in terms of estimating the EVs of the gambles. Taken together, these data fail to support the central claim of UTT, and therefore should lead the Bayesian to update his or her beliefs toward the null hypothesis – that no difference exists between unconscious and conscious deliberation. These results aside, it is instructive to evaluate how they square with other existing data on UTT. In an extensive analysis of 16 studies testing for an UT advantage, Newell and Rakow (2011) concluded that the evidence was decidedly in favor of the null when evaluated using Bayes Factors. Thus, regardless of how one interprets the findings of Ashby et al. (2011), one should be cautious in interpreting the significance of the UT advantage, if one exists.

Despite the questions surrounding earlier results, Ashby et al. (2011) found, in their first two experiments, a subtle difference between conscious and unconscious processing: when asked to select among a series of gambles, unconscious deciders selected the highest EV gamble at a higher rate than the conscious deliberators. Given that all other aspects of the study are quite similar to earlier work, the critical difference may be the use of monetary gambles as stimuli. Using these monetary gambles assumes that individuals will use weights that are explicitly provided (as probabilities), rather than developed over time. Earlier work in UTT has relied on weighting of apartment or roommate attributes that develops with experience and over time (Dijksterhuis and Nordgren, 2006). Participants are also assumed to prefer gambles based on their EV, despite substantial evidence to suggest that other factors may influence gamble preference (Tversky and Kahneman, 1981). The idea that expected utility may not be the default preference ordering adopted differentially by conscious deliberators is supported by Ashby et al.’s (2011) Experiment 3. When explicitly instructed to choose the gamble with the highest EV, they found no difference in performance for the conscious and unconscious thinkers.

Experiment 3, however, leaves the original difference between conscious deliberation without information and UT unexplained. Without explicit directions to choose the best EV gamble, the experimental manipulation used to prevent conscious deliberation produced a significantly higher rate of choosing the highest EV gamble compared to the case in which participants were free to deliberate in the absence of relevant information (Experiments 1 and 2). However, when participants are explicitly incentivized to choose the best gamble (Experiment 3), there are no differences between UT and deliberation without information, but both conditions are worse than deliberation with information. As such, some explanation for this discrepancy must exist; if the UT does indeed yield benefits in performance, then why does the effect disappear when participants are incentivized to choose the best gamble?

How can the current study be reconciled with conflicting earlier research? One possibility is that participants in the UT condition adopt a different choice strategy than participants in the deliberation condition – one in which participants attempt to recall the single best gamble, but also where memory for the numerical values is poor. In contrast, participants in the deliberation condition attempt to compute the EV of each gamble, and as a result suffer from a build-up of memory interference throughout the deliberation stage (Lassiter et al., 2009). A switch in strategy from computing EV to recall of a single best gamble would be sufficient to account for the pattern of results in all three experiments: When instructions incentivize computation of EV (Experiments 1 and 2) a deliberative strategy that involves computation would yield relatively lower errors in EV computations, but perhaps at the cost of accurately retrieving the best gamble. In contrast, a simple recall strategy without explicit computation would result in relatively better recall of the best gamble, but perhaps at the cost of greater error in EV computation. Indeed, when participants were incentivized for choosing the best gamble, but not for minimizing EV error (Experiment 3), there were no differences between the UT and deliberation without information conditions. The strategy switch hypothesis is hardly evidence for (or against, for that matter) an unconscious advantage, but merely a statement that people may do different things under different task conditions. While the central tenants of UTT have been seriously challenged by Ashby et al., we believe that their results have opened the door to potential alternative explanations for the controversial hypothesis that UT is more rational than conscious thought.
